# The Case for Early Use of Glucagon-like Peptide-1 Receptor Agonists in Obstructive Sleep Apnea Patients with Comorbid Diabetes and Metabolic Syndrome

**DOI:** 10.3390/life12081222

**Published:** 2022-08-12

**Authors:** Rizwana Sultana, Fatoumatta Sissoho, Vinod P. Kaushik, Mukaila A. Raji

**Affiliations:** 1 Division of Pulmonary Critical Care and Sleep Medicine, Department of Internal Medicine, University of Texas Medical Branch (UTMB), Galveston, TX 77555, USA; 2 Division of Geriatrics & Palliative Medicine, Department of Internal Medicine, University of Texas Medical Branch (UTMB), Galveston, TX 77555, USA

**Keywords:** diabetes, obesity, obstructive sleep apnea, GLP I-RA

## Abstract

Patients with obstructive sleep apnea (OSA) have high rates of co-occurring type 2 diabetes, hypertension, obesity, stroke, congestive heart failure, and accelerated atherosclerotic cardiovascular diseases. These conditions frequently require multiple medications, raising the risk of polypharmacy, adverse drug–drug and drug–disease interactions, decreased quality of life, and increased healthcare cost in these patients. The current review of extant literature presents evidence supporting glucagon-like peptide-1 receptor agonists (GLP-1RA) as one pharmacologic intervention that provides a “one-stop shop” for OSA patients because of the multiple effects GLP-1RA has on comorbidities (e.g., hypertension, diabetes, obesity, metabolic syndrome, and atherosclerotic cardiovascular diseases) that commonly co-occur with OSA. Examples of glucagon-like peptide-1 receptor agonists approved by the FDA for diabetes (some of which are also approved for obesity) are liraglutide, exenatide, lixisenatide, dulaglutide, semaglutide, and albiglutide. Prescribing of GLP-1RAs to address these multiple co-occurring conditions has enormous potential to reduce polypharmacy, cost, and adverse drug events, and to improve quality of life for patients living with OSA and diabetes. We thus strongly advocate for increased and early use of GLP-1RA in OSA patients with co-occurring diabetes and other cardiometabolic conditions common in OSA.

## 1. Introduction

Obstructive sleep apnea (OSA) is a chronic sleep disorder associated with multiple comorbidities (e.g., hypertension, obesity, diabetes mellitus, metabolic syndrome, and atherosclerotic cardiovascular diseases), poor quality of life, and premature death [1,2,3,4,5]. It is characterized by episodic narrowing of the upper airway during sleep [2,3,6] leading to complete or partial airway closure (apneas and hypopneas). Major risk factors of OSA include conditions which cause narrowing of airway and obesity, which is the most important risk factor. Increased adipose tissue deposition in the upper airway results in narrowing of the upper airway and increased susceptibility of collapse during sleep when muscle tone is low [3]. Apneas and hypopneas are associated with varying severity of sleep fragmentation and intermittent hypoxemia—the latter being a major factor contributing to the pathogenesis of OSA-related comorbidities [7]. Intermittent hypoxemia results in vascular endothelial cell injury, leading to significant impact on the cardiovascular system. OSA is known to have a consistent role in causing heart failure, coronary artery disease, arrhythmias, hypertension and specifically treatment-resistant hypertension [1].

Overall, obstructive sleep apnea is highly underdiagnosed, especially in non-white populations, despite having significant health and mortality impacts. [4,5,8,9]. Indeed, up to 80% of individuals with OSA remain undiagnosed despite adequate access to health care [4,8,9]. Using the 2006–2013 Medicare administrative claims data, Wickwire and colleagues showed that health care utilization is high among undiagnosed sleep apnea patients [10]. During the year prior to OSA diagnosis and relative to matched control patients, Medicare beneficiaries with sleep apnea demonstrated increased healthcare use and higher mean total annual health care costs [10]. Because of multiple associated comorbidities and the public health crisis of the rising OSA and obesity epidemic, there is an urgent need for intervention that provides a “one-stop shop” for these patients to mitigate OSA-related morbidity and mortality. The current review of literature presents evidence supporting use of glucagon-like peptide-1 receptor agonists (GLP-1RAs) as a unique pharmacologic intervention that provides such a “one-stop shop” for OSA patients because of their multiple effects on associated hypertension, diabetes mellitus, obesity, metabolic syndrome, and atherosclerotic cardiovascular diseases.

## 2. Epidemiology of Obstructive Sleep Apnea, Obesity and Diabetes

### 2.1. Obstructive Sleep Apnea and Obesity

A major risk factor for obstructive sleep apnea is obesity, but the relationship is also bidirectional [1,2,3,11,12]. The increasing rate of obesity has resulted in an increased prevalence of multiple comorbidities ranging from cardiovascular diseases to cancer, including both diabetes and OSA [2]. This phenomenon largely reflects the fact that an increase in weight gain results in upper airway narrowing. When awake and alert, patients with OSA compensate for this narrowing by increasing the activity of upper airway muscles that maintain airway patency [13]. However, during sleep or in periods of diminished alertness (from any cause including alcohol or sedative use), muscle relaxation ensues with loss of the protective effect of wakefulness thus leading to episodic collapse of the upper airway and subsequent hypoxemia and sleep fragmentation [11]. OSA is known for its significant sleep fragmentation and deprivation. Sleep deprivation results in weight gain through several mechanisms, including low leptin and high ghrelin levels, resulting in increased appetite. Another reported mechanism is increased stimulation of the renin-angiotensin-aldosterone system and aldosterone over-secretion. In addition, body mass index (BMI) is positively correlated with plasma aldosterone and angiotensinogen levels [14].

As the prevalence of obesity dramatically rises in the USA, so does the incidence of obstructive sleep apnea and type 2 diabetes mellitus. Over the past approximately 40 years, the prevalence of obesity has nearly doubled worldwide [15], with 11% of men and 15% of women aged eighteen and older considered obese in 2014. This statistic has substantially worsened with the most recent data showing a range of obesity from 27.5% to 35% among adults [16,17,18]. In the United States, the prevalence of obesity among adults aged twenty and older averages about 36%, with women at 38.3% and men 34.3% [17]. Another recent study showed more than 68% of US adults are considered overweight and 35% are obese [18]. Although sustained lifestyle modifications (diet, exercise, and stress management) are critical to reversing the obesity epidemic and its associated comorbidity of OSA and diabetes, most patients would also benefit from pharmacological intervention that treats the obesity-diabetes-OSA triad. Glucagon-like peptide-1 receptor agonists (GLP-1RAs)—approved by FDA for obesity and diabetes—is one such intervention. Therefore, GLP-1RA medication should, in addition to lifestyle interventions and CPAP therapy, be first line pharmacotherapy for OSA patients with obesity and/or diabetes.

### 2.2. Obstructive Sleep Apnea and Diabetes

Obstructive sleep apnea and diabetes commonly co-occur [19,20,21,22,23,24] with a vicious cycle of each condition worsening the severity and incidence of the other. The overall prevalence of OSA in patients with diabetes ranges from 58% to 86%, with the range reflecting the diversity of data sources, age of the sample, definition of OSA (measured versus self-report; different cut-off points for hypopneas and desaturation criteria) and sample size of different studies [23,25,26,27,28,29]. OSA is underdiagnosed in diabetes patients, with a study showing only 18% of the diabetes patients having a diagnosis of obstructive sleep apnea [30]. According to the American Diabetes Association, in 2019, about thirty-seven million Americans (approximately 11% of the US population) have diabetes, with about 1.4 million new cases diagnosed yearly [31]. Both obesity and OSA are associated with the increase in incidence and severity of diabetes [20,21,23], a statistic that is projected to worsen given the National Diabetes Statistics Report showing an approximately 88% prevalence of overweight/obesity in adults with type 2 diabetes [32]. Obesity is even highly prevalent in large proportions of individuals with type 1 diabetes mellitus (T1DM) [20]. With obesity being a major risk factor for both OSA and diabetes, it is not surprising that OSA is also associated with metabolic dysregulation, poor glucose control and increased propensity for worsening diabetes [21,22,23,24]. The intermittent hypoxemia and recurrent arousals from rest to maintain airflow have been shown to affect glucose metabolism [2]. Prolonged sleep deprivation, especially sleep duration of less than 6 h and even as a result of sleep fragmentation due to intermittent arousals, has negative effects on insulin sensitivity. Intermittent hypoxemia and arousals from sleep can lead to surges in the sympathetic nervous system which can reduce insulin-mediated glucose uptake, decrease insulin sensitivity, and impair insulin secretion [2].

The prevalence of obstructive sleep apnea ranges from 50% to 60% in persons with obesity or metabolic syndrome [22], a prevalence that dramatically increases when obesity co-occurs with diabetes mellitus [23,24]. It is therefore not a surprise that obesity-related illnesses cost per year ranges from $450–550 billion [33]. Fortunately, pharmacological interventions such as glucagon-like peptide-1 receptor agonists (GLP-1RA) that treat the obesity-diabetes-OSA triad can make substantial inroads in reversing the trends in morbidity and cost associated with obesity-related illnesses. Indeed, recent studies showed that weight loss via lifestyle modifications and pharmacotherapy can lead to resolution of OSA [34,35]. For example, a randomized controlled trial (RCT) intervention of lifestyle and other interventions that led to weight loss was associated with discontinuation of continuous positive airway pressure therapy in 62% of OSA patients at the 6-month follow-up [34,36].

### 2.3. Current Available Treatment Options for Obstructive Sleep Apnea

Multiple options are available for treatment of obstructive sleep apnea. The first line of therapy for moderate to severe sleep apnea is continuous positive airway pressure (CPAP) therapy. It is a mechanical therapy with positive airway pressure resulting in pneumatic opening of the airway and has been proven to be the most effective therapy to reduce apnea–hypopnea burden. CPAP therapy can reduce the apnea–hypopnea index (AHI) to <5 in most patients but requires tremendous effort by the patient to wear the device every night. Finding a comfortable mask is another problem faced by the patients and it can result in reduced adherence for some patients.

Patients with mild sleep apnea or patients who cannot tolerate CPAP therapy can use an oral appliance to advance the mandible in order to increase airway space. Oral appliances are proven to be effective in patients with mild to moderate sleep apnea. Available surgical options include uvulopharyngopalatoplasty or maxillomandibular advancement procedures. These surgeries are most effective in patients with craniofacial abnormalities (e.g., a hypoplastic mandible). A newer surgical treatment option is hypoglossal-nerve stimulation during sleep to move the tongue forward and open the airway [3]. There are no current medical treatment options available for obstructive sleep apnea although weight loss (a key benefit of GLP-1 receptor agonists) is recommended in all obese patients, including those using CPAP therapy [3].

### 2.4. Medications to Avoid in the Setting of Obstructive Sleep Apnea

It is important to highlight medications and substances that can potentially worsen sleep apnea through their effects on sleep architecture, muscle tone, and ventilation. These medications include, but are not limited to, central nervous system depressant drugs, propranolol or other beta-blockers, sildenafil, certain sleep agents, drugs causing weight gain, and alcohol. Central nervous system depressants such as opiates, benzodiazepines, barbiturates, and many sleep medications adversely affect ventilation during sleep by causing the upper airway to become more easily collapsible and potentiating airway closure. These substances can furthermore directly affect muscle relaxation, depress respiratory drive, arousal, and increase oxyhemoglobin desaturation [37,38]. Myorelaxants such as baclofen cause upper airway relaxation and collapse during sleep and depression of respiratory drive leading to hypoventilation. Medications that cause weight gain such as atypical antipsychotics, anticonvulsants, diabetic medications (specifically sulfonylureas and thiazolidinediones), antihistamines (especially first generation H1-antihistamines such as diphenhydramine), steroid hormones, and α- and β-adrenergic blockers should be used with caution and alternative medications should be selected, as weight gain is known to exacerbate OSA. In the case of diabetic medications, a better alternative medication in the setting of co-occurring diabetes and OSA would be metformin, which—unlike the sulfonylureas and the thiazolidinediones—does not cause weight gain and may actually promote weight loss [39,40,41].

There have been multiple proposed mechanisms of testosterone worsening sleep apnea. These mechanisms include testosterone effects on increasing the apnea–hypopnea index, prolongation of hypoxemia time, worsening sleep disorder breathing, and prolonging length of time spent with SaO_2_ < 90. [42]. A history of alcohol intake should be elicited, and patients should be discouraged from drinking alcohol as alcohol has severe deleterious effects on sleep quality and architecture. Alcohol reduces upper-airway muscle tone, delays arousal thus prolonging apnea, and increases the frequency of abnormal breathing [37,38].

### 2.5. Glucagon-Like Peptide-1 Receptor Agonist: One-Shop Drug for Obesity-Diabetes-OSA Triad

One pharmacologic intervention with potential to address diabetes mellitus, obesity and associated cardiometabolic conditions in patients with obstructive sleep apnea is glucagon-like peptide-1 receptor agonists (GLP-1RAs). The US Food and Drug Administration (FDA) approved this new class of antidiabetic medications (GLP-1RA) in 2005 and now several different preparations are available in the market (See Table 1). Table 2 summarizes some of the pathophysiological mechanisms for the GLP-I-receptor agonist benefits in the setting of OSA and comorbidities including the potential salutary effects of GLP-1 RA in moderating systemic inflammation and reversing endothelial dysfunction. The rest of the article will provide a summary of the evidence that supports the basis of our recommendation that patients with co-occurring OSA, obesity with or without diabetes should be considered for GLP-1RA as first line medication of choice given its salutary effects on the obesity-diabetes-obstructive sleep apnea triad [19,25].

### 2.6. GLP-1RA: Effects on Co-Occurring Cardiometabolic Diseases in OSA Patients

Patients with OSA have high rates of comorbidities, which are known to improve in the setting of GLP-1RA medication use [19,43,44]. Evidence from two double-blinded randomized controlled trial (RCT) studies also showed that glucagon-like peptide-1 receptor agonist use is associated with improvement in apnea–hypopnea index (AHI), independent of GLP-1RA weight-loss effects and associated decrease in body mass index (BMI) [45,46]; this suggests a direct yet-to-be determined mechanism on various pathways involved in OSA pathogenesis. Blackman and colleagues chose patients with moderate to severe obstructive sleep apnea who were unwilling to use continuous positive airway pressure therapy and randomized them to either Liraglutide therapy along with diet control with five hundred fewer calories or placebo group with diet control only [46]. Average body mass index was 39.1. After 32 weeks of treatment, reduction in the AHI was greater in the Liraglutide group with higher weight loss and also greater reduction in glycosylated hemoglobin (hemoglobin A1c-HbA1c) [46]. This study proves the superiority of glucagon-like peptide-1 receptor agonist medications in treating diabetes mellitus, obstructive sleep apnea, and obesity at the same time.

### 2.7. GLP-1RA: Effects on Co-Occurring Obesity in OSA Patients

The evidence for glucagon-like peptide-1 receptor agonists in promoting weight loss has been well documented and forms the basis of the Food and Drug Administration (FDA) approval of several GLP-1 receptor agonists as medications for weight loss in those with BMI > 35 or BMI > 30 with a serious comorbidity. [19,47,48]. A randomized controlled trial conducted by Makgos et al. showed that a 5% weight loss led to improvement in adipose tissue, liver, and muscle insulin sensitivity, and β-cell function [49]. GLP1 medications have been shown to not only reduce hemoglobin AIc (HbA1c) and blood glucose levels, but they are proven to be effective for weight loss. Yaribeygi and colleagues published a comprehensive study on multiple molecular mechanisms and pathways by which GLP-1RA medications reduce HbA1c and improve glycemic control [50]. GLP-1RAs are known stimulators for pancreatic beta cells and secrete insulin during periods of elevated levels of blood glucose after meals. Yaribeygi also discussed the role of GLP-1RAs in reducing oxidative stress, which causes insulin resistance, hence improving insulin sensitivity in peripheral tissues [50]. GLP-1RAs also reduce insulin resistance by reducing proinflammatory mediators. Other mechanisms include suppressing glucagon secretion and slowing the gastric emptying [50].

Other researchers also described the multiple biological pathways by which GLP-1RAs improve the obesity-diabetes dyad: increasing satiety and delaying of gastric emptying leading to promotion of weight loss along with a decrease in hemoglobin A1C and without increase in hypoglycemic episodes [19,51,52]. Currently, the American Diabetes Association (ADA), European Association for the Study of Diabetes (EASD), American Association of Clinical Endocrinologists (AACE), and American College of Endocrinology (ACE) recommend GLP-1RAs to be used as adjunct medications when adequate glucose control is not achieved with lifestyle interventions (including weight reduction) and a non-insulin oral drug [39,40,41]. Others recommended adding GLP-1RAs to basal insulin for better HbA1c control [53]. We the authors recommend that patients with co-occurring OSA, obesity with or without diabetes be considered, from the first clinic contact, for GLP-1RA as the first line medication of choice given its salutary effects on this triad [19].

Currently, pharmacotherapy is recommended as an adjunct to lifestyle modification (diet, exercise) for individuals with a body mass index of at least 30 kg/m^2^, or at least 27 kg/m^2^ with comorbidity such as OSA or atherosclerotic cardiovascular diseases (ASCVD). [19,44,45,53]. Multiple FDA-approved anti-obesity medications exist (see semaglutide and liraglutide in Table 1), but their prescribing and utilization rates are low [54,55], a reflection of a myriad of factors including low awareness of obesity and related illnesses as a public health crisis, fat-shaming and biases by clinicians when interacting with patients with obesity, suboptimal reimbursement with substantial out-of-pocket costs to patients, suboptimal content on obesity management during undergraduate and graduate medical education, negative views of obesity drugs based on bad reputation/safety concerns in the past, and lack of confidence in the efficacy of anti-obesity drugs among patients and their caregivers [56,57,58].

## 3. Clinical Implications and Conclusions

In patients—especially those aged 65 and older—with obstructive sleep apnea and co-occurring type 2 diabetes mellitus, obesity and other cardiometabolic conditions, we suggest a discussion during the initial clinic visit about both the lifestyle approaches and pharmacologic interventions with glucagon-like peptide-1 receptor agonists as evidence-informed approaches to improve obstructive sleep apnea-related apnea–hypopnea index (AHI), control diabetes without hypoglycemic risks, lower weight, blood pressure and lipids, and reduce subsequent risk of stroke and heart attack [19]. The evidence for the recommendations come from observational and clinical trial data. The multiple effects GLP-1RAs have on obesity and ASCVD—a reflection of the multisystemic roles of GLP-1 receptor functions in the setting of diabetes and OSA—can potentially lead to a reduction of polypharmacy and cost, amelioration of OSA-related cardiometabolic derangements, and enhancement of OSA-related quality of life indicators [19,25,45,46,47,48,49,50,51,52,53]. The evidence hitherto presented underscores the usefulness of GLP-1RAs in reducing overall medication burden and the urgent need for large randomized, controlled, clinically comparative trials of GLP-1RA in the setting of co-occurring OSA, diabetes and/or obesity or the triad. Table 2 summarizes the beneficial effects of GLP-1RAs on OSA. Given the high percentage of underdiagnosed sleep apnea in patients with diabetes and/or obesity, early initiation of GLP-1RA medication therapy can have superior benefits and reduce the need for polypharmacy especially in older individuals with multimorbidity. We strongly advocate that medical providers consider these medications very early in the OSA disease process to prevent complications. Education of all clinicians as well as patients with OSA along with a campaign to raise public awareness are needed to ensure an early discussion between patients and clinicians regarding the possibility of initiation of treatment with GLP-1RA medications—a crucial step in managing the common co-occurring metabolic syndromes, atherosclerotic cardiovascular diseases and obesity in patients living with OSA.

## Figures and Tables

**Table 1:** Currently available glucagon-like peptide-1 receptor agonists in the USA.

Drug	Brand Name	Dosage
Exenatide	Byetta	By injection twice daily
Exenatide extended release (ER)	Bydureon	Taken by injection weekly
Liraglutide	Victoza	Taken by injection daily
Semaglutide	Ozempic	Taken by injection weekly
Semaglutide	Rybelsus	Taken by mouth daily
Albiglutide	Tanzeum	Taken by injection weekly
Dulaglutide	Trulicity	Taken by injection weekly
Lixisenatide	Adlyxin	Taken by injection daily
∗ Tirzepatide	Mounjaro	Taken by injection weekly

∗ Dual (Glucose-dependent Insulinotropic Polypeptide (GIP)/Glucagon-Like Peptide (GLP1) Receptor Agonist.

**Table 2:** Putative Mechanisms of GLP1 RA agonist Beneficial Effects in OSA Patients from references.

⇓ Weight
⇓ Perineck/Peri-oropharyngeal Soft Tissue
⇓OSA-associated ASCVD: CVA, Hypertension, Arrythmias, Heart Failure, Myocardial Infarction, Sudden Cardiac Death
⇓ Metabolic Syndrome
⇓ Hemoglobin A1C and Diabetes Complications
⇓ Systemic Inflammation
⇓ Metabolic Dysregulation
⇓ Endothelial Dysfunction
⇓ Vascular Dementia
⇓ Risk of Delirium
⇓ Depression

## Data Availability

Not applicable.

## References

[1] Rana D., Torrilus C., Ahmad W., Okam N.A., Fatima T., Jahan N. (2020). Obstructive Sleep Apnea and Cardiovascular Morbidities: A Review Article. Cureus.

[2] Aurora R.N., Punjabi N.M. (2013). Obstructive sleep apnoea and type 2 diabetes mellitus: A bidirectional association. Lancet Respir. Med..

[3] Veasey S.C., Rosen I.M. (2019). Obstructive Sleep Apnea in Adults. N. Engl. J. Med..

[4] Lee Y.C., Chang K.Y., Mador M.J. (2022). Racial disparity in sleep apnea-related mortality in the United States. Sleep Med..

[5] Howrey B.T., Peek M.K., Raji M.A., Ray L.A., Ottenbacher K.J. (2012). Self-reported sleep characteristics and mortality in older adults of mexican origin: Results from the Hispanic established population for the epidemiologic study of the elderly. J. Am. Geriatr. Soc..

[6] West S.D., Turnbull C. (2018). Obstructive sleep apnoea. Eye.

[7] Dewan N.A., Nieto F.J., Somers V.K. (2015). Intermittent hypoxemia and OSA: Implications for comorbidities. Chest.

[8] Lee W., Nagubadi S., Kryger M.H., Mokhlesi B. (2008). Epidemiology of Obstructive Sleep Apnea: A Population-based Perspective. Expert Rev. Respir. Med..

[9] Kapur V., Strohl K.P., Redline S., Iber C., O’Connor G., Nieto J. (2002). Underdiagnosis of sleep apnea syndrome in U.S. communities. Sleep Breath..

[10] Wickwire E.M., Tom S.E., Vadlamani A., Diaz-Abad M., Cooper L.M., Johnson A.M., Albrecht J.S. (2020). Older adult US Medicare beneficiaries with untreated obstructive sleep apnea are heavier users of health care than matched control patients. J. Clin. Sleep Med..

[11] Drager L.F., Togeiro S.M., Polotsky V.Y., Lorenzi-Filho G. (2013). Obstructive sleep apnea: A cardiometabolic risk in obesity and the metabolic syndrome. J. Am. Coll. Cardiol..

[12] Mergen H., Altındağ B., Zeren U.Z., Karasu K.I. (2019). The Predictive Performance of the STOP-Bang Questionnaire in Obstructive Sleep Apnea Screening of Obese Population at Sleep Clinical Setting. Cureus.

[13] Schwab R.J., Pasirstein M., Pierson R., Mackley A., Hachadoorian R., Arens R., Pack A.I. (2003). Identification of upper airway anatomic risk factors for obstructive sleep apnea with volumetric magnetic resonance imaging. Am. J. Respir. Crit. Care Med..

[14] Rodrigues G.D., Fiorelli E.M., Furlan L., Montano N., Tobaldini E. (2021). Obesity and sleep disturbances: The “chicken or the egg” question. Eur. J. Intern. Med..

[15] World Health Organization (WHO) (2014). Global Status Report on Noncommunicable Diseases 2014.

[16] Apovian C.M. (2016). Obesity: Definition, comorbidities, causes, and burden. Am. J. Manag. Care.

[17] Ogden C.L., Carroll M.D., Fryar C.D., Flegal K.M. (2015). Prevalence of Obesity Among Adults and Youth: United States, 2011–2014. NCHS Data Brief.

[18] Flegal K.M., Carroll M.D., Ogden C.L., Curtin L.R. (2010). Prevalence and trends in obesity among US adults, 1999–2008. JAMA.

[19] Onoviran O.F., Li D., Toombs S.S., Raji M.A. (2019). Effects of glucagon-like peptide 1 receptor agonists on comorbidities in older patients with diabetes mellitus. Ther. Adv. Chronic Dis..

[20] Polsky S., Ellis S.L. (2015). Obesity, insulin resistance, and type 1 diabetes mellitus. Curr. Opin. Endocrinol. Diabetes Obes..

[21] Lam J.C., Mak J.C., Ip M.S. (2012). Obesity, obstructive sleep apnoea and metabolic syndrome. Respirology.

[22] Resta O., Foschino-Barbaro M.P., Legari G., Talamo S., Bonfitto P., Palumbo A., De Pergola G. (2001). Sleep-related breathing disorders, loud snoring and excessive daytime sleepiness in obese subjects. Int. J. Obes. Relat. Metab. Disord..

[23] Foster G.D., Sanders M.H., Millman R., Zammit G., Borradaile K.E., Newman A.B., Sleep Ahead Research Group (2009). Obstructive sleep apnea among obese patients with type 2 diabetes. Diabetes Care.

[24] Valencia-Flores M., Orea A., Castaño V.A., Resendiz M., Rosales M., Rebollar V., Bliwise D.L. (2000). Prevalence of sleep apnea and electrocardiographic disturbances in morbidly obese patients. Obes. Res..

[25] Pamidi S., Tasali E. (2012). Obstructive sleep apnea and type 2 diabetes: Is there a link?. Front. Neurol..

[26] Resnick H.E., Redline S., Shahar E., Gilpin A., Newman A., Walter R., Study S.H.H. (2003). Diabetes and sleep disturbances: Findings from the Sleep Heart Health Study. Diabetes Care.

[27] Einhorn D., Stewart D.A., Erman M.K., Gordon N., Philis-Tsimikas A., Casal E. (2007). Prevalence of sleep apnea in a population of adults with type 2 diabetes mellitus. Endocr. Pract..

[28] Laaban J.P., Daenen S., Léger D., Pascal S., Bayon V., Slama G., Elgrably F. (2009). Prevalence and predictive factors of sleep apnoea syndrome in type 2 diabetic patients. Diabetes Metab.

[29] Aronsohn R.S., Whitmore H., Van Cauter E., Tasali E. (2010). Impact of untreated obstructive sleep apnea on glucose control in type 2 diabetes. Am. J. Respir. Crit. Care Med..

[30] Heffner J.E., Rozenfeld Y., Kai M., Stephens E.A., Brown L.K. (2012). Prevalence of diagnosed sleep apnea among patients with type 2 diabetes in primary care. Chest.

[31] American Diabetes Association (2015). Statistics about Diabetes. https://www.diabetes.org/resources/statistics/statistics-about-diabetes.

[32] Centers for Disease Control and Prevention (2017). Estimates of Diabetes and Its Burden in the United States. National Diabetes Statistics Report.

[33] Cawley J., Meyerhoefer C., Biener A., Hammer M., Wintfeld N. (2015). Savings in Medical Expenditures Associated with Reductions in Body Mass Index Among US Adults with Obesity, by Diabetes Status. Pharmacoeconomics.

[34] Carneiro-Barrera A., Amaro-Gahete F.J., Guillén-Riquelme A., Jurado-Fasoli L., Sáez-Roca G., Martín-Carrasco C., Ruiz J.R. (2022). Effect of an Interdisciplinary Weight Loss and Lifestyle Intervention on Obstructive Sleep Apnea Severity: The INTERAPNEA Randomized Clinical Trial. JAMA Netw. Open.

[35] Carneiro-Barrera A., Díaz-Román A., Guillén-Riquelme A., Buela-Casal G. (2019). Weight loss and lifestyle interventions for obstructive sleep apnoea in adults: Systematic review and meta-analysis. Obes. Rev..

[36] Tuomilehto H., Seppä J., Uusitupa M., Tuomilehto J., Gylling H., Group K.S.A. (2013). Weight reduction and increased physical activity to prevent the progression of obstructive sleep apnea: A 4-year observational postintervention follow-up of a randomized clinical trial. JAMA Intern. Med..

[37] Strollo P.J., Rogers R.M. (1996). Obstructive sleep apnea. N. Engl. J. Med..

[38] Larive L.L., Tisdale J.E., Miller D.A. (2005). Sleep disorders. Drug-Induced Diseases.

[39] Inzucchi S.E., Bergenstal R.M., Buse J.B., Diamant M., Ferrannini E., Nauck M., Matthews D.R. (2015). Management of hyperglycemia in type 2 diabetes, 2015: A patient-centered approach: Update to a position statement of the American Diabetes Association and the European Association for the Study of Diabetes. Diabetes Care.

[40] Handelsman Y., Bloomgarden Z.T., Grunberger G., Umpierrez G., Zimmerman R.S., Bailey T.S., Zangeneh F. (2015). American association of clinical endocrinologists and american college of endocrinology—Clinical practice guidelines for developing a diabetes mellitus comprehensive care plan—2015. Endocr. Pract..

[41] Garber A.J., Abrahamson M.J., Barzilay J.I., Blonde L., Bloomgarden Z.T., Bush M.A., Davidson M.H. (2015). AACE/ACE comprehensive diabetes management algorithm 2015. Endocr. Pract..

[42] Jullian-Desayes I., Revol B., Chareyre E., Camus P., Villier C., Borel J.C., Joyeux-Faure M. (2017). Impact of concomitant medications on obstructive sleep apnoea. Br. J. Clin. Pharmacol..

[43] Lévy P., Kohler M., McNicholas W.T., Barbé F., McEvoy R.D., Somers V.K., Pépin J.L. (2015). Obstructive sleep apnoea syndrome. Nat. Rev. Dis. Primers.

[44] McEvoy R.D., Antic N.A., Heeley E., Luo Y., Ou Q., Zhang X., Mediano O., Chen R., Drager L.F., Liu Z. (2016). CPAP for Prevention of Cardiovascular Events in Obstructive Sleep Apnea. N. Engl. J. Med..

[45] Amin R.S., Naong S.N., Szczesniak R.V. (2015). Treatment of obstructive sleep apnea with glucagon like peptide-1 receptor agonist. Am. J. Respir. Crit. Care Med..

[46] Blackman A., Foster G.D., Zammit G., Rosenberg R., Aronne L., Wadden T., Mignot E. (2016). Effect of liraglutide 3.0 mg in individuals with obesity and moderate or severe obstructive sleep apnea: The SCALE Sleep Apnea randomized clinical trial. Int. J. Obes..

[47] Ryan D., Acosta A. (2015). GLP-1 receptor agonists: Nonglycemic clinical effects in weight loss and beyond. Obesity.

[48] Shah M., Vella A. (2014). Effects of GLP-1 on appetite and weight. Rev. Endocr. Metab. Disord..

[49] Magkos F., Fraterrigo G., Yoshino J., Luecking C., Kirbach K., Kelly S.C., Klein S. (2016). Effects of Moderate and Subsequent Progressive Weight Loss on Metabolic Function and Adipose Tissue Biology in Humans with Obesity. Cell Metab..

[50] Yaribeygi H., Sathyapalan T., Sahebkar A. (2019). Molecular mechanisms by which GLP-1 RA and DPP-4i induce insulin sensitivity. Life Sci..

[51] Gardner H., Hamdy O. (2020). Oral GLP1 Analog: Where Does the Tide Go?. Clin. Med. Insights Endocrinol. Diabetes.

[52] Maselli D.B., Camilleri M. (2021). Effects of GLP-1 and Its Analogs on Gastric Physiology in Diabetes Mellitus and Obesity. Adv. Exp. Med. Biol..

[53] Diamant M., Nauck M.A., Shaginian R., Malone J.K., Cleall S., Reaney M., de Vries D., Hoogwerf B.J., MacConell L., Wolffenbuttel B.H.R. (2014). Glucagon-like peptide 1 receptor agonist or bolus insulin with optimized basal insulin in type 2 diabetes. Diabetes Care.

[54] Garvey W.T., Mechanick J.I., Brett E.M., Garber A.J., Hurley D.L., Jastreboff A.M., Nadolsky K., Pessah-Pollack R., Plodkowski R. (2016). American association of clinical endocrinologists and american college of endocrinology comprehensive clinical practice guidelines for medical care of patients with obesity. Endocr. Pract..

[55] Simon R., Lahiri S.W. (2018). Provider practice habits and barriers to care in obesity management in a large multicenter health system. Endocr. Pract..

[56] Thomas C.E., Mauer E.A., Shukla A.P., Rathi S., Aronne L.J. (2016). Low adoption of weight loss medications: A comparison of prescribing patterns of antiobesity pharmacotherapies and SGLT2s. Obesity.

[57] Baum C., Andino K., Wittbrodt E., Stewart S., Szymanski K., Turpin R. (2015). The Challenges and Opportunities Associated with Reimbursement for Obesity Pharmacotherapy in the USA. Pharmacoeconomics.

[58] Kaplan L.M., Golden A., Jinnett K., Kolotkin R.L., Kyle T.K., Look M., Dhurandhar N.V. (2018). Perceptions of Barriers to Effective Obesity Care: Results from the National ACTION Study. Obesity.

